# Machine Learning for CRISPR-Based Diagnostics

**DOI:** 10.3390/ijms27125485

**Published:** 2026-06-17

**Authors:** Haniel Siqueira Mortagua Walflor, Lia Carolina Soares Medeiros

**Affiliations:** Cellular Biology Laboratory, Instituto Carlos Chagas, Fiocruz Paraná, Rua Professor Algacyr Munhoz Mader 3775, Cidade Industrial de Curitiba (CIC), Curitiba 81350-010, Paraná, Brazil; haniel.smw@gmail.com

**Keywords:** CRISPR, diagnostics, machine learning, deep learning, guide RNA design, signal classification, foundation models

## Abstract

CRISPR-based diagnostics now detect viral, bacterial, and cancer-associated nucleic acids with sensitivities approaching quantitative PCR; however, their translation to decentralized care rests on computational design and interpretation that current datasets cannot sustain. Pandemic-era Cas12a assays reached 95% positive predictive agreement against reverse transcription quantitative PCR (RT-qPCR) at 10 copies/μL, and deep neural networks now design Cas13 detection assays spanning 1933 vertebrate-infecting viruses, ranking candidate guides at Spearman correlations of 0.69 to 0.84 across internal and external validation. Generative deep-learning systems improve single-nucleotide discrimination two- to three-fold, computer vision classifies lateral flow outputs at 96.5% accuracy, and multi-biomarker fusion reaches an area under the receiver operating characteristic curve (AUC) of 0.998 in lung cancer detection. These results mask a narrow data foundation. Cas13a guide prediction still draws from a single screening library of 19,209 guide–target pairs, Cas12a has one published diagnostic model, and signal classifiers almost uniformly validate on single-site cohorts. This review synthesizes mechanistic constraints, predictive and generative models, and point-of-care classifiers, and maps the path beyond this data ceiling. Evolutionary pretraining on RNA corpora and lab-in-the-loop agents that convert model failure into targeted data acquisition define the route forward.

## 1. Introduction

Emerging viral pathogens, antimicrobial-resistant bacteria, and the rising global cancer burden demand diagnostic technologies that combine molecular precision with deployment flexibility [1,2]. Established nucleic acid amplification methods achieve the requisite sensitivity and specificity, but their dependence on thermal cycling equipment, centralized laboratories, and trained operators restricts access in resource-limited settings where diagnostic need is often greatest [3,4]. CRISPR-Cas systems offer a fundamentally different architecture for molecular detection, one that couples programmable target recognition with enzymatic signal amplification under isothermal conditions [5].

The diagnostic utility of CRISPR derives from collateral cleavage, a phenomenon wherein target recognition by the Cas effector triggers indiscriminate nuclease activity against reporter substrates [6,7]. The single-effector architecture of Class 2 systems, wherein a single multidomain protein mediates both target recognition and cleavage, simplifies biochemical reconstitution relative to multi-component Class 1 systems and thereby favors deployment in point-of-care settings [8,9]. This property underlies the DETECTR (DNA Endonuclease-Targeted CRISPR Trans Reporter) and HOLMES (One-HOur Low-cost Multipurpose highly Efficient System) platforms, which employ Cas12a for DNA target detection [7,10], and the SHERLOCK (Specific High-sensitivity Enzymatic Reporter unLOCKing) platform, which uses Cas13 for RNA targets [6]. These systems detect viral, bacterial, and cancer-associated nucleic acids with sensitivities approaching quantitative PCR when coupled with isothermal pre-amplification [7,11,12]. Clinical validation during the COVID-19 pandemic confirmed these capabilities, with DETECTR-based SARS-CoV-2 detection achieving 95% positive predictive agreement and 100% negative predictive agreement against reverse transcription quantitative PCR (RT-qPCR) at a limit of detection of 10 copies/μL in approximately 30 to 40 min using a lateral flow readout format [13].

CARMEN (Combinatorial Arrayed Reactions for Multiplexed Evaluation of Nucleic acids) extends these single-target assays to multiplexed detection, achieving greater than 4500 crRNA-target pairs on a single array, differentiating 169 human-associated viruses, and reducing reagent costs by more than 300-fold through microfluidic miniaturization [14]. The CRISPRD platform combines *Aap*Cas12b, *Tcc*Cas13a, and *Hhe*Cas13a in a one-pot loop-mediated isothermal amplification (LAMP)-coupled format for simultaneous human papillomavirus (HPV) 16/18 detection, achieving a limit of detection of 10 copies/μL with 100% specificity [15]. Translation studies are accelerating: a recent preprint reports that the BADLOCK (Bacterial and Antimicrobial Resistance Detection by SHERLOCK) platform identified bloodstream-infection pathogens and antimicrobial-resistance genes from positive blood cultures with 97.6% reaction-level accuracy across clinical and mock specimens in a one-pot format compatible with point-of-care (POC) development [16].

A central challenge limiting CRISPR-based diagnostic (CRISPR-Dx) development is not the detection mechanism itself but the identification of effective guide RNAs [17]. Guide RNA (crRNA) efficacy depends on biophysical factors, including target accessibility, mismatch tolerance, and sequence recognition motifs, that resist simple prediction. In Cas12 and Cas13 systems, the crRNA alone directs target recognition, unlike the single-guide RNA (sgRNA) of Cas9 systems [18]. Cas9 requires fusion of the crRNA with a trans-activating crRNA (tracrRNA) to form a functional sgRNA, whereas Cas12 and Cas13 effectors are guided solely by the crRNA and do not require tracrRNA for activity [7,19].

High-throughput screens reveal that approximately 97% (287 of 296) of crRNAs produce functional Cas13a activation under optimized conditions [20], yet distinguishing high-activity sequences from adequate ones demands screening dozens to hundreds of candidates per target, and multiplexed pathogen panels render this search combinatorially intractable. Machine learning (ML) and deep learning (DL) now address this bottleneck across both guide design and point-of-care signal interpretation, but generalization beyond narrow training distributions remains an open challenge rooted in the biological complexity of Cas effector systems. We frame this transition as a shift from hardware (CRISPR biochemistry) to software (computational design), a framing that makes the data-foundation gap visible across both predictive guide design and signal classification. We begin by discussing the mechanistic constraints that govern CRISPR-based detection, because they define both the input features that predictive models must encode and the failure modes that clinical deployment must overcome.

## 2. Mechanistic Constraints in CRISPR-Based Detection

### 2.1. Molecular Recognition and Target Discrimination

**Figure 1:**
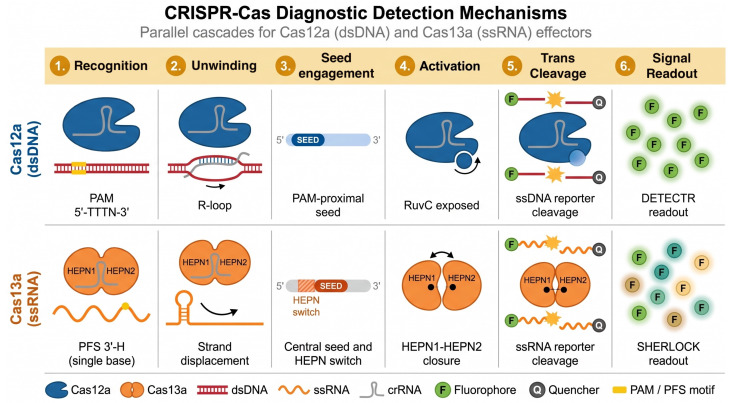
CRISPR-Cas diagnostic detection mechanisms. Parallel six-stage cascades for Cas12a (dsDNA detection, blue, top row) and Cas13a (ssRNA detection, orange, bottom row). The six stages proceed left to right across the columns. (1) Recognition: Cas12a engages a T-rich PAM ($5^{\prime }$-TTTN-$3^{\prime }$) on target dsDNA [7,21,22], whereas Cas13a recognizes a single-base PFS ($3^{\prime }$-H, non-G) on target ssRNA [19,24]. (2) Unwinding/access: Cas12a forms an R-loop through a DNA-unwinding equilibrium that gates RuvC activation [25]; Cas13a actively displaces target secondary structure to expose the protospacer [26]. (3) Seed engagement: the seed is PAM-proximal in Cas12a but central in Cas13a, which also contains a distinct HEPN-switch zone; seed mismatches reduce Cas13a affinity 47-fold while HEPN-switch mismatches block activation without disrupting binding [24] (see Figure 2 for ortholog-resolved position ranges). (4) Activation: Cas12a undergoes a two-step allosteric transition exposing RuvC [27]; Cas13a closes HEPN1 toward HEPN2, converging catalytic residues into a composite active site [28]. (5) Trans-cleavage: Cas12a cleaves ssDNA reporters at $k_{cat}∼1$–$4\;s^{-1}$, versus ∼0.04–0.05 s^−1^ for dsDNA reporters; Cas13a cleaves ssRNA reporters at $k_{cat}∼27\;s^{-1}$ [29]. (6) Signal readout: liberated fluorophores (F) separate from quenchers (Q), generating detectable fluorescence signal (DETECTR for Cas12a [7]; multiplexed channels for SHERLOCK [6,11]). Legend: dsDNA (red double ribbon), ssRNA (orange wave), crRNA (gray ribbon), fluorophore F (green dot), quencher Q (dark gray dot), PAM/PFS recognition motif (yellow).

Sensitive and specific CRISPR assays demand careful navigation of the biological and kinetic constraints governing Cas nuclease activity (Figure 1). Sequence recognition motifs impose the most fundamental constraint on guide design. Cas12a requires a T-rich protospacer adjacent motif (PAM; $5^{\prime }$-TTTN-$3^{\prime }$, though TTTV sites (V = A, C, or G) are preferred because TTTT shows reduced activity [21]) on target dsDNA to initiate cleavage [7,22]. RNA-targeting Cas13 enzymes instead recognize a protospacer flanking sequence (PFS), though this requirement varies by ortholog. *Lsh*Cas13a activity requires a $3^{\prime }$-H PFS (H = A, C, or T) [19], whereas *Lwa*Cas13a shows only a weak $3^{\prime }$-H PFS preference [23]. Mechanistic studies of *Lsh*Cas13a revealed that a “G” at the PFS position does not prevent RNA binding but blocks the conformational transition required for catalytic activation, effectively decoupling target binding from nuclease activation [24].

Kinetic constraints control target discrimination independently of sequence recognition. In Cas12a, discrimination proceeds through a DNA unwinding equilibrium that functions as a mismatch-sensing checkpoint before nuclease activation [25]. This checkpoint underlies Cas12a’s sensitivity to PAM-distal mismatches, as the enzyme assesses the stability of the unwound DNA state before licensing RuvC nuclease activity. Structural dynamics at the PAM-adjacent region also influence off-target binding. Single-molecule studies demonstrated that DNA flexibility at the PAM + 1 position dictates off-target Cas12a binding, with flexibility at PAM + 2 and PAM + 3 further augmenting this effect [30].

Cas12a activation follows a two-step kinetic mechanism in which rapid target binding is followed by a slower allosteric transition [27]. Bases within the PAM region, target topology, and activator length all modulate activation kinetics, and canonical PAM sequences are not strictly required for trans-cleavage.

RNA targets are cleaved by Cas12a approximately 860-fold slower than double-stranded DNA targets ($k_{obs}=0.0008\;\min^{-1}$ versus $0.69\;\min^{-1}$) [27], with correspondingly slower trans-activation. Ortholog selection therefore strongly influences assay performance, particularly under POC conditions where thermal control may be limited. A systematic comparison of Cas12a orthologs showed that *Ts*Cas12a maintains robust activity at 25 °C, achieving 23-fold greater sensitivity than *Lb*Cas12a at room temperature (LoD: 98 versus 2300 copies/μL at 60 min) [31].

The improved sensitivity of *Ts*Cas12a arises primarily from differences in activation rate constant ($k_{act}$) rather than steady-state catalytic parameters. *Ts*Cas12a exhibits a $k_{act}$ of 2.7 min^−1^, approximately 6-fold higher than that of *Lb*Cas12a (0.45 min^−1^). One-pot recombinase polymerase amplification (RPA)-CRISPR assays using *Ts*Cas12a achieved 100% sensitivity and 100% specificity for HPV-16 detection across 16 clinical samples without external heating, demonstrating that ortholog-specific kinetic optimization can enable equipment-free diagnostics [31].

Substrate identity at the PAM-proximal (Pp) region further constrains Cas12a activation, with direct implications for assay design and RNA detection. Systematic characterization of split-activator systems revealed that the first 10 to 12 nucleotides of the spacer, corresponding to the seed region, must be DNA to trigger trans-cleavage in *Lb*Cas12a and *Er*Cas12a [32]. This strict DNA requirement persists even when the PAM-distal (Pd) portion of the activator consists of RNA, establishing a molecular basis for differential substrate tolerance across the crRNA spacer. Activator length also constrains detection sensitivity. Lengths of 14 nucleotides or fewer produce no detectable trans-cleavage, and 16-nucleotide activators show 50- to 70-fold reduced activity compared to full-length 20-nucleotide sequences. These constraints motivated the development of SAHARA (Split-Activator Hybrid Assay for RNA Analysis), which exploits the Pd region’s tolerance for RNA substrates by pairing a short synthetic DNA oligonucleotide at the Pp region with target RNA binding at the Pd region, enabling reverse-transcription-free and amplification-free RNA detection with picomolar limits of detection [32].

**Figure 2:**
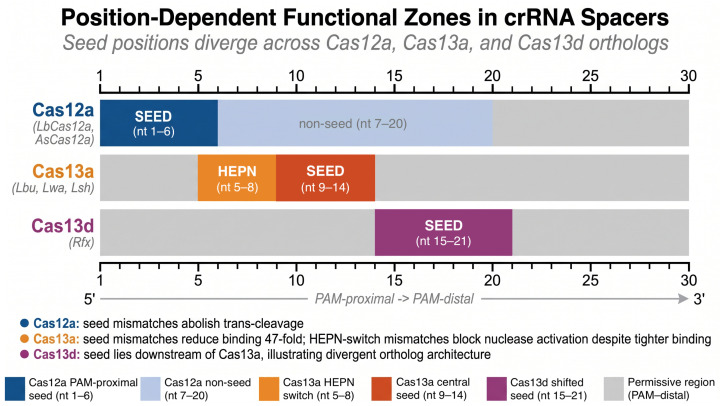
Position-dependent functional architecture of crRNA spacers across three diagnostically relevant Cas effectors. Schematic representation of functionally distinct zones within crRNA spacer sequences (positions 1–30, numbered from the $5^{\prime }$ end). *Lb*Cas12a and *As*Cas12a concentrate mismatch sensitivity within a PAM-proximal seed region (nt 1–6), where single mismatches severely impair cleavage activity. *Lbu*Cas13a exhibits a bipartite architecture comprising a HEPN-nuclease switch (nt 5–8), where mismatches permit 10–100-fold tighter target binding while abolishing nuclease activation, and a central seed region (nt 9–14) where mismatches cause up to 47-fold decreases in binding affinity [24]. *Rfx*Cas13d displays a shifted seed architecture (nt 15–21) that diverges from Cas13a orthologs [34], demonstrating that seed position varies substantially across the Cas13 family. The divergent seed architectures establish that guide design rules require ortholog-specific calibration and cannot be generalized across effector families.

Target accessibility and mismatch tolerance shape the interaction between crRNA spacer and target protospacer [5]. crRNA configurations and target-site secondary structures modulate nuclease activity [23,33]. Cas13 enzymes preferentially cleave single-stranded RNA [19], and target structures occluding the protospacer region inhibit activity [20,26]. Cas13a overcomes this competition via strand displacement, a key kinetic factor in target recognition [26]. Mismatch sensitivity further modulates detection through position-dependent effects. In Cas13a, a central seed region spanning spacer nucleotides 9 to 14 governs stable target RNA binding, with mismatches at these positions causing a 47-fold decrease in affinity that effectively abrogates complex formation [24]. An adjacent Higher Eukaryotes and Prokaryotes Nucleotide-binding (HEPN)-nuclease switch region at nucleotides 5 to 8 gates catalytic activation independently of binding; mismatches here paradoxically permit 10- to 100-fold tighter binding while completely abolishing nuclease activity, thereby decoupling target recognition from signal generation [24]. Crystal structures of *Lbu*Cas13a attribute this decoupling to a 6.5 to 9.6 Å HEPN1-HEPN2 conformational closure upon target binding [28]. Cas13d exhibits a distinct seed architecture, with mismatch sensitivity concentrated at spacer nucleotides 15 to 21 rather than the central region observed in Cas13a [34] (Figure 2).

Ortholog-specific cleavage preferences expand the design space beyond guide–target interactions and directly influence reporter design. Functional characterization of ten Cas13a homologs revealed two orthogonal subfamilies distinguished by crRNA recognition and ssRNA cleavage specificity. The *Lbu*Cas13a-like subfamily preferentially cleaves at uridine residues, whereas the *Lba*Cas13a-like subfamily favors adenosine [35]. Sensitivity varies by up to $10^{7}$-fold across homologs, providing a functional basis for multiplexed detection using spectrally orthogonal reporters.

Structural and biochemical analyses further showed that many orthologs preferentially cleave motifs containing uridine or adenosine, although the precise sequence preference differs among enzymes. *Lwa*Cas13a is uridine-preferring and was implemented with an AU reporter in SHERLOCKv2, *Lbu*Cas13a preferentially cleaves poly-U substrates, *Psm*Cas13b favors GA motifs, and *Cca*Cas13b targets UC sequences [11,35]. This orthogonal cleavage specificity enabled SHERLOCKv2, which uses four spectrally distinct reporter channels matched to different Cas13 orthologs for simultaneous multiplexed detection [11]. Because the optimal reporter dinucleotide tracks the chosen effector, guide and reporter form a joint design target, and reporter identity becomes a model feature that current sequence-only guide-design architectures omit.

**Figure 3:**
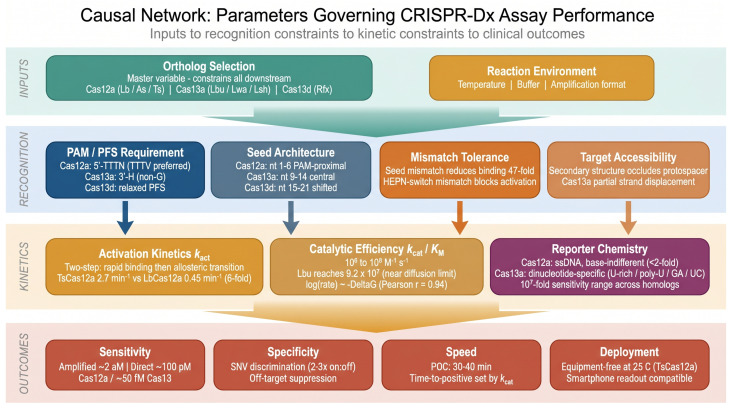
Causal relationships among the parameters governing CRISPR-Dx assay performance. Four-tier cascade illustrating how two independent inputs (top) constrain a tier of four recognition constraints and a subsequent tier of three kinetic constraints (middle), which together determine the diagnostic outcome dimensions (bottom). Ortholog selection functions as the master variable, directly constraining seed region architecture through ortholog-specific positional sensitivity (nt 1–6 for Cas12a, nt 9–14 for Cas13a; Figure 2), reporter chemistry, and temperature-dependent activation kinetics where $k_{act}$ varies 6-fold between *Ts*Cas12a (2.7 min^−1^) and *Lb*Cas12a (0.45 min^−1^), with *Ts*Cas12a achieving 23-fold greater sensitivity at room temperature [31]. Reporter requirements diverge fundamentally between effector families. Cas13 orthologs exhibit dinucleotide-specific cleavage preferences spanning a $10^{7}$-fold sensitivity range across homologs [35], whereas Cas12a cleaves ssDNA with minimal sequence selectivity (≤2-fold base preference), permitting generic reporter designs [29]. Seed region identity determines mismatch tolerance profiles that govern single-nucleotide variant discrimination, with seed mismatches causing up to 47-fold decreases in binding affinity [24]. Target accessibility influences activation kinetics through strand displacement requirements, with trans-cleavage rate scaling exponentially with unwinding free energy (ΔG; Pearson r=0.94) [36]. These causal dependencies establish that guide RNA design cannot be optimized independently of ortholog choice, reporter selection, and target secondary structure (Table 1). These recognition and kinetic parameters constitute the engineering half of the two-pillar framework that this review develops, with the second pillar (deployment validation) addressed in Section 4.2.

PAM and PFS requirements, seed region identity, target accessibility, mismatch tolerance landscapes, and ortholog-specific cleavage preferences collectively define a multidimensional constraint space that directs CRISPR-based detection. These parameters are not independent but rather form an interconnected network in which seed region composition affects mismatch sensitivity, target secondary structure modulates the kinetic contribution of strand displacement, and ortholog selection determines temperature-dependent activation rates, reporter substrate preferences, and seed architecture itself (Figure 3; Table 1). Empirical optimization of such interdependent constraints rapidly becomes intractable, making computational approaches essential for navigating the design space. The kinetic parameters that determine how rapidly and efficiently these molecular recognition events translate into detectable signal impose additional boundaries on assay performance.

### 2.2. Catalytic Kinetics and Detection Sensitivity

Molecular recognition determines whether detection occurs, whereas kinetic and thermodynamic parameters govern how sensitively and reproducibly it performs. Amplification-coupled assays using RPA or LAMP routinely achieve attomolar limits of detection (LoD) because pre-amplification generates sufficient target copies to saturate Cas activation [39]. Amplification-free formats rely instead on the intrinsic catalytic efficiency of Cas enzymes, imposing distinct limitations on both achievable sensitivity and computational optimization strategies. Format selection and reaction condition optimization therefore depend on these kinetic and thermodynamic constraints.

Cas13a activation proceeds through sequential target RNA binding, cis-cleavage, and high-turnover trans-RNase activity. Target binding follows second-order kinetics and occurs rapidly at diagnostic concentrations, whereas cis-cleavage follows first-order kinetics approximately 5000-fold slower than trans-cleavage ($k_{obs}\approx 0.005\;s^{-1}$ versus $k_{cat}\approx 27\;s^{-1}$) [6,11,29]. The resulting active ribonucleoprotein complex catalyzes multiple substrate cleavages per activation event.

Trans-cleavage follows Michaelis–Menten kinetics. Writing the trans-cleavage rate *v* in terms of the target-activated effector concentration $\left[E^{*}\right]$ and the reporter substrate concentration [S] gives

(1) $$v=\frac{k_{cat}\;\left[E^{*}\right]\;\left[S\right]}{K_{M}+\left[S\right]},$$

where $k_{cat}$ is the turnover number and $K_{M}$ the Michaelis constant. Amplification-free assays operate in the substrate-limited regime ($\left[S\right]\ll K_{M}$), where Equation (1) reduces to $v\approx \left(k_{cat}/K_{M}\right)\;\left[E^{*}\right]\;\left[S\right]$, so the catalytic efficiency $k_{cat}/K_{M}$, rather than $k_{cat}$ alone, sets achievable sensitivity. Measured efficiencies span $10^{6}$ to $10^{8}\;M^{-1}\;s^{-1}$ depending on substrate, and *Lbu*Cas13a reaches $9.2\times 10^{7}\;M^{-1}\;s^{-1}$, approaching the diffusion-controlled limit [29]. Reporter substrate composition and length modulate this efficiency by more than an order of magnitude. Polyuridine substrates (U10, U20) yield $k_{cat}$ values of 273–320 s^−1^ for *Lbu*Cas13a, whereas the RNaseAlert probe achieves only 17.1 s^−1^, an approximately 19-fold reduction that masks the enzyme’s full catalytic potential [29]. Catalytic efficiency $k_{cat}/K_{M}$ therefore defines the optimization objective for diagnostic guide design, and its substrate dependence sets the floor on the limit of detection any amplification-free assay can promise.

Cas12a trans-cleavage, by contrast, discriminates substrates primarily by structure rather than sequence, with ssDNA outperforming dsDNA by 60- to 70-fold in $k_{cat}$ and base identity contributing ≤2-fold variation [29]. Although this structural selectivity eliminates the need for ortholog-specific dinucleotide matching required by Cas13 systems, reporter format still modulates catalytic output. For instance, 10-mer ssDNA probes achieve $k_{cat}$ values of 2.77–3.83 s^−1^ compared with 1.02–1.30 s^−1^ for the commercial DNaseAlert reporter, representing a 2- to 4-fold improvement achievable through probe length and composition alone [29].

Cas12a faces additional thermodynamic constraints that slow its kinetics relative to Cas13a [29]. Following target-specific dsDNA recognition and cis-cleavage, Cas12a undergoes conformational rearrangement that exposes its nonspecific ssDNase activity. This activation step couples to a DNA unwinding equilibrium checkpoint, the same mismatch-sensing mechanism that governs target discrimination, in which the free-energy change (ΔG) of nucleic acid unwinding controls reaction rates [25,36].

For single-stranded substrates, the logarithm of the trans-cleavage rate increases linearly with the negative unwinding free energy,

(2) $$\ln k_{\mathrm{trans}}=-\alpha \;\Delta G_{\mathrm{unwind}}+\beta \;\left(r=0.94\right),$$

where α and β are empirical constants with α>0, indicating an exponential dependence of the trans-cleavage rate on unwinding energetics. For double-stranded substrates, this relationship instead becomes parabolic [36]. Strand separation energy therefore defines the rate-limiting barrier for activation and constrains overall trans-cleavage efficiency.

These kinetic and thermodynamic parameters translate directly into detection sensitivity. Comparative measurements across Cas12 and Cas13 orthologs have correlated enzymatic rate constants with achievable LoDs in amplification-free assays [40]. Successful detection requires cleavage of approximately 0.1% of fluorescent reporters to exceed background, although this threshold varies with reaction conditions [40].

Given typical catalytic efficiencies ($k_{cat}/K_{M}$ of $10^{5}$ to $10^{6}\;M^{-1}\;s^{-1}$) and practical assay timescales of 30 to 60 min, the theoretical LoD for amplification-free detection falls in the picomolar range under standard conditions. Amplification-free detection at approximately 100 copies/μL has been achieved using combined crRNAs together with sensitive smartphone-based fluorescence detection [41]. By contrast, amplification-coupled assays routinely detect single-digit copies per microliter. For applications targeting low viral loads or circulating biomarkers, this difference determines whether upstream amplification is required.

Independent validation studies have revealed systematic inconsistencies in published kinetic parameters for CRISPR enzymes. A Michaelis–Menten-based analytical framework, incorporating three consistency rules derived from species conservation, maximum reaction velocities, and reaction time scales, showed that nearly all published studies reporting CRISPR enzyme kinetics violate at least two of these rules [42]. Subsequent analysis identified substantial violations of mass conservation and chemical kinetics, with some studies reporting signal intensities up to 90-fold higher than the theoretical maximum permitted by the available reporter molecules [43]. Because models inherit these errors from the kinetic measurements that train them, benchmark standards and self-consistency screens gate the data quality on which reproducible machine learning depends.

Subsequent investigations identified unit conversion errors as a primary cause of these discrepancies, prompting published errata for several seminal studies [7]. Consequently, turnover rates ($k_{cat}$) of 1000 to 5000 s^−1^ reported in early work overestimate true values by at least two orders of magnitude. Selection bias may have further propagated similarly inflated values through the subsequent literature.

Trans-cleavage kinetics depend strongly on Cas homolog selection, crRNA sequence, target characteristics, and reporter chemistry [44]. Compilations in the literature report corrected *Lb*Cas12a $k_{cat}$ values ranging from 0.022 to 17 s^−1^ across activator types and experimental conditions [42,44], whereas side-by-side substrate-capture measurements place ssDNA reporter turnover near 1–4 s^−1^ [29]. This spread illustrates the substantial variability in reported parameters.

Monte Carlo propagation of experimental uncertainties, including pipetting error, inner-filter effect, and calibration bias, through the classical Michaelis–Menten framework demonstrated that even carefully measured $k_{cat}$ and $K_{M}$ values are accurate only within a twofold range [45]. Fluorescent reporter degradation introduces a nonenzymatic background process that imposes an additional limit on assay sensitivity [46].

An augmented kinetics model incorporating a first-order degradation term showed that reporter decay can dominate signal evolution at low target concentrations, explaining anomalous signal increases in negative controls and defining a revised theoretical LoD. Engineering guidelines have since established benchmarks for kinetic data quality across Cas systems, with catalytic efficiency ($k_{cat}/K_{M}$) emerging as the most robust metric for comparing enzyme performance because it remains more consistent than either parameter measured in isolation [38].

Target binding specificity and catalytic efficiency jointly define the CRISPR-Dx optimization landscape, which machine learning addresses by learning the composite sequence-to-performance mapping from empirical data.

## 3. Applications of ML in CRISPR-Dx

**Figure 4:**
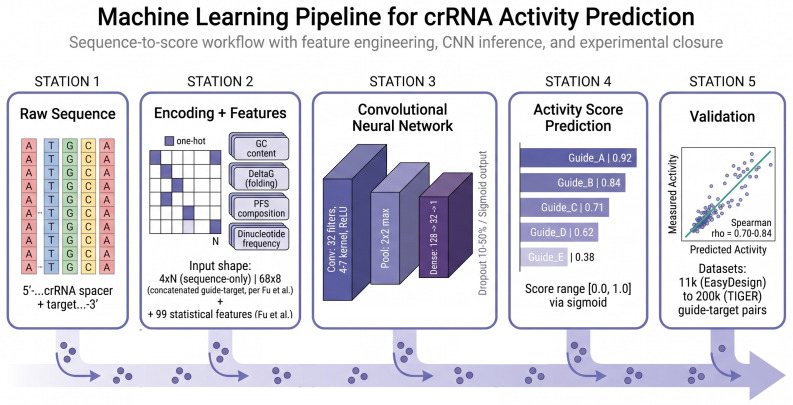
Machine learning pipeline for crRNA activity prediction. Five-station workflow illustrating the computational approach to guide RNA optimization. Raw Sequence: Raw DNA/RNA sequences enter the pipeline. Encoding and Features: Sequences undergo one-hot encoding to generate nucleotide matrices (4 × N), sometimes combined with derived features such as GC content, folding free energy (ΔG), PFS composition, and dinucleotide frequencies. Convolutional Neural Network: Representative CNN-based models process these features through convolutional blocks, pooling, and fully connected layers, but published implementations differ in filter number, kernel size, hidden-layer width, activation function, and dropout. Activity Score Prediction: Sigmoid activation produces activity scores (0–1 scale) enabling guide ranking and prioritization. Validation: A predicted-versus-measured scatter plot benchmarks ranked guides against experimental activity, achieving Spearman ρ correlations of 0.69–0.84 with measured activity (Table 2). Training corpora for these pipelines remain confined to single Cas orthologs (Cas13a or Cas12a) and guide-target counts of 11,000 to 200,000 (Section 3.1) [51].

Machine learning, and deep learning in particular, has shifted CRISPR-Dx from empirical optimization toward computationally guided assay design (Figure 4). This transition builds upon an established body of computational crRNA design tools, from rule-based scoring systems and early ML methods to off-target scoring frameworks developed initially for gene editing applications [47]. Deep networks automate hierarchical feature extraction, capturing complex nonlinear relationships that traditional ML models miss [48]. Representation learning allows these models to process raw biological sequences directly, discovering latent regulatory patterns without human-defined features [49].

Computational models now predict crRNA activity, specificity, and off-target potential from sequence alone [50], while computer vision algorithms classify readout signals, distinguish true positives from noise, and enable quantitative target detection.

### 3.1. Precision Assay Design and Guide RNA Optimization

Because mismatches at different spacer positions exert separable effects on binding versus activation (Section 2), nucleotide identity at defined positions carries predictive information, a property well suited to DL architectures that learn positional dependencies directly from sequence data.

Building on these structural foundations, convolutional neural network (CNN) architectures emerged as the dominant approach for predictive crRNA modeling. The DeepCpf1 model, an early CNN trained on approximately 15,000 Cas12a (Cpf1) target sequences to predict gene editing efficiency, demonstrated that deep learning could extract sequence motifs predictive of guide activity, achieving Spearman correlations of 0.60 to 0.83 across validation datasets (its sequence-only variant, Seq-deepCpf1, achieves lower correlation) [50]. This early result positioned CNNs as superior to classical ML approaches (random forests, support vector machines) for guide activity prediction, though the limited training data constrained generalization to novel pathogen targets. Concurrent work explored two-dimensional convolutional architectures operating on dinucleotide-encoded sequence matrices, with 5 × 5 kernels for on-target activity prediction and 7 × 7 kernels for off-target specificity, showing that spatial representations of local sequence context could capture patterns at multiple scales [56].

The transition from individual guide testing to massively parallel screening transformed the data foundations for ML. Screening over 24,000 Cas13d crRNAs identified key sequence features governing RNA-target recognition, including a functional seed region at nucleotides 15 to 21 [34]. Although this work focused on therapeutic Cas13d applications, we hypothesize that the general principles of seed region sensitivity and mismatch tolerance shape diagnostic Cas13a design rules. The Cas13d seed region lies downstream of the position characterized in Cas13a structural studies, and differing PFS requirements further suggest that design rules require ortholog-specific calibration. These large-scale screens also revealed the first indications of generalization challenges, as guides optimized for one pathogen family often underperformed when applied to divergent targets with different sequence compositions.

The ADAPT framework constituted a paradigm shift from isolated activity prediction to integrated assay design. Combining deep neural network predictions with combinatorial optimization, the Activity-informed Design with All-inclusive Patrolling of Targets (ADAPT) system produced guide sets maximizing viral genomic coverage [37]. Trained on over 19,209 guide–target pairs and calibrated to achieve 0.975 precision at a defined classification threshold, ADAPT designed CRISPR-Cas13 assays for 1933 vertebrate-infecting viruses within hours and produced a SARS-CoV-2 SHERLOCK assay detecting 10 RNA copies/μL [57], matching or exceeding established DETECTR and SHERLOCK benchmarks. By incorporating amplification primer design into its workflow (though primer selection relied on conventional heuristics rather than learned models), ADAPT demonstrated that computational design could achieve pandemic-scale pathogen coverage while maintaining diagnostic sensitivity. Subsequent clinical validation extended this approach beyond viral targets, with the BADLOCK (Bacterial and Antimicrobial Resistance Detection by SHERLOCK) platform employing ADAPT-designed guides to achieve 97.6% reaction-level accuracy across 2224 reactions targeting nine bacterial species and four antimicrobial-resistance genes from positive blood cultures [16], confirming cross-pathogen transferability of ML-guided design principles.

As datasets expanded further, the field confronted persistent generalization challenges. Hybrid CNN-long short-term memory (LSTM) architectures for Cas13 systems showed promise by capturing both local sequence features and longer-range positional dependencies [54], yet experimental validation quantified performance degradation across biological contexts. The DeepCas13 model, trained on Cas13d knockdown screening data from Wessels et al. [34] and integrating convolutional and recurrent layers with sequence and structure features, achieved an area under the receiver operating characteristic curve (AUC) of 0.87 on its held-out *CD46/55/71* five-fold cross-validation set, while an independent evaluation on an HT29 circular RNA (circRNA) test set yielded an area under the precision–recall curve (AUPR) of 0.57 [54], illustrating performance shifts across datasets and target classes. The absence of standardized benchmark datasets and evaluation metrics deepened these concerns, making meaningful comparison across published approaches difficult [58]. The gap between reported training metrics and real-world performance widened whenever models encountered sample matrices, reagent batches, or environmental conditions not represented in training data.

The same research group that first characterized Cas13d guide design rules from 24,000 crRNAs [34] subsequently scaled to approximately 200,000 crRNAs with the TIGER (Targeted Inhibition of Gene Expression via gRNA design) framework, mapping activity landscapes across the Cas13d spacer and revealing that G and C nucleotides in the seed region contribute strongly to guide activity while mismatches are generally better tolerated than indels [55]. By integrating non-sequence features such as crRNA folding free energy and mismatch tolerance, TIGER achieved improved generalization across orthologous Cas13d enzymes. We hypothesize that learned representations transfer to diagnostic Cas13a applications when retrained on diagnostic-specific data. Complementary biochemical characterization using the RNA-CHAMP platform profiled over 10,000 target RNA variants to map binding and cleavage determinants across diverse sequence contexts, providing orthogonal validation of computationally identified features [59]. Dataset size tracked generalization capacity, with models trained on more than 100,000 guide–target pairs outperforming those trained on smaller cohorts across held-out datasets.

Whereas the preceding approaches characterize sequence–activity relationships from experimental data, generative models invert this process to create novel guides with desired properties. The BADGERS (Building Artificial Diagnostic Guides by Exploring Regions of Sequences) system, the first generative approach in diagnostic guide design, couples a conditional Wasserstein generative adversarial network with activation maximization (WGAN-AM), employing ADAPT’s CNN activity predictor as its fitness function to evaluate candidate guides [53]. BADGERS generated artificial Cas13a guides bearing intentional mismatches to natural targets, improving sensitivity by 10 to 20% and achieving on-target-to-off-target signal ratios 2–3-fold higher than those of natural-sequence guides when discriminating clinically relevant single-nucleotide polymorphisms (SNPs). Among the mechanistic design rules that emerged was the tag-adjacent mismatch (TAM), illustrating how generative deep learning can discover biochemical heuristics rather than merely replicate patterns observed in training data.

The EasyDesign CNN adapted ADAPT’s architectural framework to the Cas12a system, training on 11,496 experimentally validated detection cases from 34 bacterial and viral pathogens and achieving Spearman correlations of 0.79 to 0.84 (mean 0.81) across five-fold cross-validation [52]. Reusing the ADAPT architecture confirmed that CNN frameworks designed for Cas13a can be retrained for Cas12a diagnostics, though the model was trained from scratch rather than through transfer learning. The model generalized beyond its training distribution, designing effective guides for previously unseen pathogens including monkeypox virus. A dual-branch neural network for Cas13a systems, trained and evaluated on the ADAPT dataset [37], combined one-hot sequence encodings with 99 statistically derived features (mismatch frequency, protospacer flanking sequence composition), achieving an AUC of 0.882 and area under the precision–recall curve (AUPR) of 0.975 while outperforming single-branch CNNs [51]. Integrating handcrafted statistical features with deep-learning architectures enhanced interpretability while supporting rational crRNA design. The dual-branch strategy parallels results from Cas13d knockdown models, where derived biophysical features provided information complementary to learned sequence representations, pointing toward convergence between therapeutic and diagnostic design approaches.

**Figure 5:**
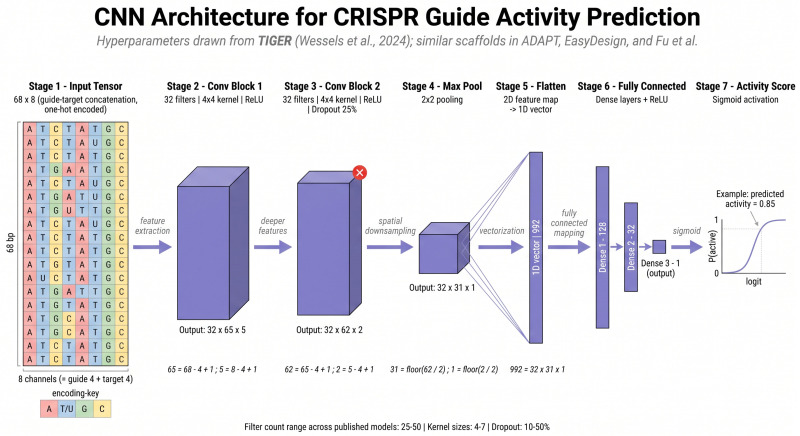
Common architectural themes in CRISPR guide activity prediction models. Architectural choices across DeepCpf1 [50], ADAPT [37], EasyDesign [52], TIGER [55], and the dual-branch network of Fu et al. [51] converge on a shared CNN scaffold, here drawn as a single seven-stage pipeline. Stage 1 is the input tensor, a 68 × 8 one-hot encoding (A, T, G, C indicated by color) that concatenates the guide and target sequences (four channels each). Stages 2 and 3 are two convolutional blocks, each with 32 filters, a 4 × 4 kernel, and ReLU activation; the second block adds 25% dropout regularization. Stage 4 applies 2 × 2 max pooling, Stage 5 flattens the resulting feature map into a 1D vector, and Stage 6 passes it through fully connected dense layers with ReLU activation. Stage 7 produces an activity score between 0.0 and 1.0 via sigmoid activation. Across published models the corresponding hyperparameters vary (filter counts of 25–50, kernel sizes of 4–7, and dropout of 10–50%) alongside the reported ranking performance summarized in Table 2, but cross-study comparisons remain limited by differences in Cas effector, training set size, and validation design.

Comparing input encoding and architecture choices across these models (Figure 5) reveals a common scaffold of one-hot input, 1D or 2D convolutional blocks, and optional feature concatenation. Divergence appears where long-range dependencies matter (hybrid CNN-LSTM) [60] or where handcrafted features are available (dual-branch designs). Per-model specifics, including kernel size, dropout rate, and layer depth, are summarized in Table 2.

Performance comparison across these models confirms several trends (Table 2), though the lack of common benchmarks noted above complicates direct comparison [58]. Simpler models with well-curated features nonetheless match deeper networks while retaining greater interpretability and lower susceptibility to overfitting on smaller datasets.

Documented limitations accompany these advances [60,61]. Although several frameworks incorporate off-target prediction, including TIGER (Pearson *r* = 0.76 for off-target activity) and BADGERS (which maximizes on-target versus off-target discrimination), most ML tools optimize primarily for on-target activity without systematic exclusion of cross-reactive binding sites in complex clinical specimens. Complementary genomics-based specificity validation tools, such as the PathoGD pipeline performing k-mer exclusion and pangenome analysis, filter ML-optimized guides for potential off-target binding to non-pathogenic sequences [21]. No published architecture encodes the DNA unwinding equilibrium that gates Cas12a activation, even though this checkpoint is the clearest physics constraint distinguishing trans-cleavage from cis-cleavage prediction [25]. The data concentration, single-ortholog training, and generalization challenges underlying these limitations structure the community-benchmarking agenda developed in Section 4.1. Even when guide design succeeds, the resulting signal must still be interpreted reliably under field conditions, a problem that has driven a parallel computational effort in automated readout.

### 3.2. Automated Readout and Signal Classification

**Figure 6:**
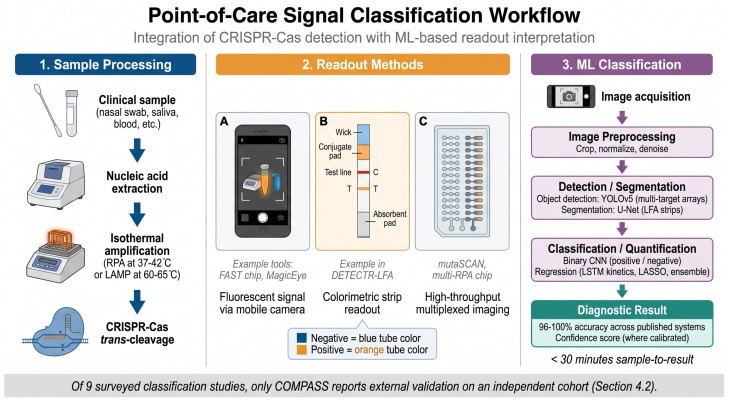
POC signal classification workflow. Integration of CRISPR-Cas detection with machine-learning-based readout interpretation across three stages. Sample Processing (left): Clinical samples undergo nucleic acid extraction, isothermal amplification (RPA/LAMP), and CRISPR-Cas detection. Readout Methods (center): three readout modalities are supported. (A) Fluorescence signal captured by a mobile camera (examples: FAST chip, MagicEye); (B) colorimetric lateral flow assay (LFA) strips with test (T) and control (C) lines; (C) high-throughput microfluidic-array imaging (example: mutaSCAN, multi-RPA chip). Tube readouts follow a shared color key (negative = blue, positive = orange). ML Classification (right): Smartphone-based image acquisition followed by preprocessing, CNN/YOLO detection (YOLOv5 for multi-target, U-Net for segmentation), object detection, segmentation, and classification stages. Across the cited systems, automated readout achieves 96–100% accuracy, with sub-second mobile classification and total assay time determined by the upstream chemistry.

While deep learning has transformed crRNA optimization, signal interpretation poses a distinct challenge for CRISPR-Dx (Figure 6), particularly in POC settings, where fluorescence and lateral flow assay (LFA) outputs require reliable automated reading. Visual assessment of colorimetric or fluorescent signals near detection limits introduces substantial inter-operator variability and systematic bias. ML and computer vision approaches now automate readout classification and quantification, enabling objective and portable diagnostic workflows.

Computer vision provided the first automated alternative to manual binary classification, with lightweight algorithms achieving reliable performance across diverse readout formats. By 2022, the funnel-adapted sensing tube (FAST) chip showed that simple models could interpret visual signals without heavy computational resources [62]. The FAST format reached a LoD of 1 fM, and a linear kernel model analyzing histogram and grayscale features distinguished positive from negative samples with near-perfect accuracy. The MagicEye smartphone application refined this approach through a two-stage deep-learning pipeline [63], employing a single-shot MultiBox detector to localize reaction tubes and a binary CNN for colorimetric interpretation. MagicEye achieved 100% accuracy on test datasets, outperforming human visual readings at low target concentrations where unaided assessment failed, though validation was limited to synthetic SARS-CoV-2 samples and a single smartphone model. Scaling from single-tube assays to high-throughput arrays shifted the challenge from simple classification to complex spatial recognition. The YOLOv5 (You Only Look Once, version 5) object detection algorithm, applied to a portable multi-RPA-CRISPR chip, enabled automated screening of genetically modified organisms [64]. This trained YOLOv5-S model achieved a mean average precision (mAP@0.5) of 0.996 and allowed simultaneous and automated detection of CaMV35S and NOS targets with a LoD of 1 aM. A 2024 study addressed spatial complexity in high-throughput microfluidics systems by integrating a pretrained Fast R-CNN (Fast Region-based Convolutional Neural Network), implemented in Detectron2, into the mutaSCAN system [65]. Processing up to 96 samples, this deep-learning pipeline identified reaction units and classified SARS-CoV-2 variants (wild type vs. Omicron) with 100% prediction accuracy for reaction times exceeding 10 min.

Even in formats vulnerable to physical artifacts, such as LFAs, computer vision maintains classification accuracy. Semantic segmentation models adapted to overcome variable lighting and strip alignment issues achieved 96.5% classification accuracy with inference times under 0.2 s using a U-Net segmentation model and lightweight MnUV3 network (a MobileNetV3-derived architecture optimized for mobile deployment) trained on approximately 3000 smartphone images [66]. Across POC implementations, deep-learning interpretation consistently improves LFA sensitivity and specificity while reducing readout time below three minutes [67].

Beyond binary classification, regression frameworks extract continuous quantitative data from assay signals. A CRISPR-Cas12a DETECTR SARS-CoV-2 assay integrated with a smartphone reader used logistic regression to translate pixel intensity ratios into a quantitative fluorescence score that correlated with RT-qPCR cycle threshold values (mean AUC = 0.93) [68], though validation was limited to 115 clinical samples from a single hospital site. Non-linear ML algorithms extend quantification into ultrasensitive regimes, as shown by a Random Forest Regression algorithm analyzing microdroplet images for meat adulteration testing that achieved a LoD of 0.00001% genomic DNA mixing ratio by processing parameters such as droplet area and intensity [69]. A 2025 study developed a Least Absolute Shrinkage and Selection Operator (LASSO) regression model for a dual-mode microRNA (miRNA)-33 assay to handle ratiometric spectral signals [70]. Analyzing the ratio of fluorescence emissions ($F_{558}/F_{430}$), the model achieved an $R^{2}$ of 0.9951 and a robust performance-to-deviation ratio of 14.34, with accurate quantification down to 4.6 fM.

Ensemble approaches further improve quantitative precision in complex environmental matrices. A stacked ensemble learning model combining Random Forest, XGBoost, and Ridge Regression monitored organophosphorus pesticide degradation using colorimetric features extracted from smartphone images [71]. The model achieved a coefficient of determination ($R^{2}$) of 0.9985, demonstrating that ensemble methods can suppress noise introduced by heterogeneous environmental matrices. Temporal analysis provides additional advantages over endpoint measurements, as reaction kinetics contain information that static measurements discard. A 2025 systematic evaluation of ML strategies for reaction kinetics analysis compared regression-based methods with a long short-term memory (LSTM) recurrent neural network applied to time-series fluorescence data [72]. The LSTM achieved 87% sensitivity, 100% specificity, and 92.14% overall accuracy while reducing analysis time by approximately 40% relative to fluorescence-magnitude baselines. Notably, learned temporal features such as slope, inflection point, and time-to-threshold transferred more effectively across reaction conditions than static intensity thresholds.

The ML requirements for CRISPR-Dx readout diverge between binary classification (positive/negative) and quantitative measurement (target concentration). Binary classification tasks can be addressed with relatively simple architectures, as demonstrated by linear kernel models and lightweight CNNs achieving near-perfect accuracy on test datasets [62,63]. These models primarily learn to threshold signal intensity while maintaining robustness under variable conditions of noise and illumination. Quantitative diagnostics, by contrast, present greater challenges, requiring models to learn the full relationship between signal and target concentration. Regression frameworks must account for the nonlinear kinetics of CRISPR reactions, dynamic range limitations, and transitions between pre-amplification and post-saturation regimes. LSTM-based approaches that analyze full reaction kinetics are particularly effective for quantitative applications, as they extract temporal features such as reaction velocity, curve shape, and time-to-threshold that correlate more reliably with initial target concentration than endpoint intensity measurements alone [72] (Table 3).

Pre-analytical variability (extraction efficiency, inhibitors, matrix effects) further erodes robustness, since several classifiers are trained or tuned on synthetic targets, purified samples, or small single-laboratory datasets [62,66,72]. The training data gaps, environmental dependencies, and single-site validation patterns underlying these deployment constraints structure the multi-site validation agenda developed in Section 4.2.

Multi-biomarker fusion extends beyond single-signal classification, using ML to combine readouts from multiplexed CRISPR assays into composite diagnostic scores. In complex diseases such as cancer, single biomarkers often lack sufficient specificity or dynamic range, whereas a composite molecular signature achieves higher discriminative power [74]. A 2025 iontronic CRISPR-Cas12a biosensor coupled with a Random Forest classifier diagnosed lung cancer in a 100-sample cohort from combined signals of methylated biomarkers (*MALAT1* and *HOTAIR*) [73]. Neither marker alone was strongly diagnostic, with individual AUCs of 0.651 for *MALAT1* and 0.794 for *HOTAIR*, whereas the Random Forest integrating both signals achieved a test-set AUC of 0.998 at 96.7% accuracy. The FEVOR assay for colorectal cancer [75] extends this principle to circulating biomarkers. CRISPR-Cas13a quantifies four extracellular-vesicle-derived miRNAs, and a Linear Discriminant Analysis model fuses these signals into a composite score that achieved AUC = 0.940 (sensitivity 91.7%; specificity 92.8%) in independent validation, outperforming carcinoembryonic antigen and other clinical biomarkers. The COMPASS platform for non-small cell lung cancer [74] uses Cas12a trans-cleavage to quantify nine aptamer-bound serum proteins, with a neural network selecting an optimal four-marker subset (the FOON panel) that achieved AUC = 0.954 and 89.6% accuracy in external validation. All three platforms implement end-to-end workflows in which CRISPR generates multiplexed quantitative signals and ML optimizes their integration for clinical classification.

## 4. Challenges and Future Directions

**Figure 7:**
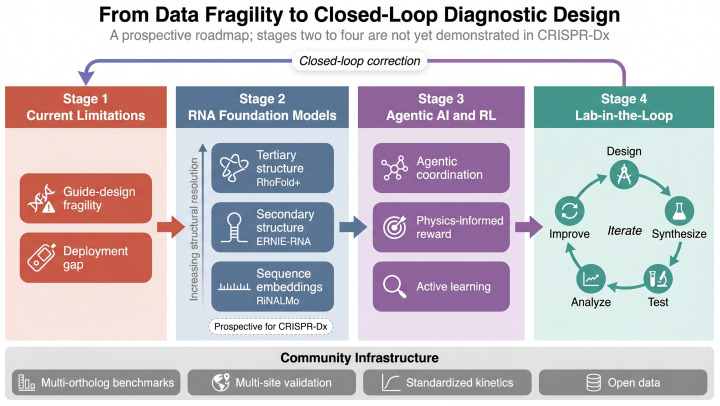
From data fragility to closed-loop diagnostic design: a roadmap for AI-driven CRISPR-Dx. Four interconnected stages illustrate how the field can progress from current limitations to autonomous assay optimization. Stages two to four are prospective and have not yet been demonstrated for CRISPR-Dx guide design. Current Limitations (left, red): Guide design suffers from data concentration around a single Cas13a screening library and a single Cas12a diagnostic model, while signal classification is constrained by single-site validation (eight of nine classification-focused entries), absent confidence calibration, and uncharacterized environmental variability. Both limitations share a common root cause, namely narrow training data that cannot capture clinical variability. RNA Foundation Models (center-left, slate blue): Three models span a hierarchy of increasing structural resolution. RiNALMo (650M parameters, 36 million non-coding RNA sequences) provides general sequence embeddings capturing latent structural motifs [76]. ERNIE-RNA (86M parameters) injects thermodynamic base-pairing priors to achieve secondary structure prediction with cross-family F1 scores approximately twice those of RiNALMo [77]. RhoFold+ combines language model pretraining (on approximately 23.7 million RNA sequences) with MSA co-evolutionary signals for end-to-end tertiary structure prediction and protospacer occlusion detection [78]. Experimental validation of the pretrain-then-finetune paradigm comes from a modified nanoGPT that predicted ribosomal RNA thermostability mutations, confirmed by a 3-fold activity increase at 65 °C [79]. Agentic AI and RL (center-right, purple): Agentic coordination couples large language model planning with bioinformatics tool integration and closes the hypothesis-experiment cycle through autonomous analysis. Physics-informed reinforcement learning encodes PAM/PFS constraints, unwinding free energy (ΔG), temperature-dependent activation kinetics ($k_{act}$), and reporter-ortholog co-optimization as reward components. Active learning directs wet-lab experiments toward guide-target pairs with high ensemble disagreement, converting model uncertainty into targeted data acquisition [80]. Lab-in-the-Loop (right, teal): Iterative design-synthesize-test-analyze-improve cycles enable progressive distribution expansion; in an adjacent therapeutic-antibody domain, four rounds raised the hit rate for 3-fold-improved binders from 2% to 21% while model accuracy rose from Spearman ρ=0.35 to 0.54 [81]. (Bottom strip): Community infrastructure prerequisites, including multi-ortholog benchmarks, multi-site validation matrices, standardized kinetic data, and open data repositories, must be established before foundation model or RL advances can be distinguished from artifacts of data scale or institutional protocol.

Four interconnected stages separate current limitations from closed-loop diagnostic design (Figure 7). The narrow-data foundation manifests in both predictive guide design (Section 4.1) and POC signal classification (Section 4.2). Foundation models compensate for this scarcity through pretrained representations (Section 4.3), and agentic systems convert model failure into targeted data acquisition (Section 4.4).

### 4.1. Data Constraints in Predictive Guide Design

A single dataset dominates Cas13a guide prediction, creating systemic fragility across the field. The Metsky et al. library introduced in Section 3.1 trains both ADAPT and downstream models including BADGERS [37]. CARMEN enables high-throughput Cas13a screening but requires specialized microfluidic infrastructure (chip fabrication, droplet generation, and custom imaging) that most laboratories lack [14], whereas Cas12a has no equivalent platform, forcing reliance on labor-intensive plate-based workflows. Cas12a diagnostic prediction fares worse. As of this writing, EasyDesign stands alone as the only published ML model for Cas12a trans-cleavage activity prediction [52]. Other Cas12a architectures target cis-cleavage for genome editing [50], and their applicability to diagnostic contexts has not been tested because trans-cleavage kinetics obey distinct enzymatic turnover rules [40]. Training set sizes spanning approximately 11,000 to over 200,000 guide–target pairs confound direct comparison [52,55], and apparent performance differences may reflect data scale rather than algorithmic innovation.

The generalization failures are not architectural failures. They are data failures. When a model performs well on development data and collapses on a new pathogen or cell line, the learned sequence–activity relationships have memorized dataset-specific correlations rather than biochemical principles that transfer. In practice, a model trained on one Cas ortholog under one experimental protocol will not reliably predict guide performance under different conditions without fresh calibration data. Current training sets cover a sliver of the parameter space that clinical diagnostics actually requires, spanning few pathogens, one or two Cas orthologs, and narrow windows of reaction conditions. Off-target specificity in clinical specimens remains an additional unresolved gap (Section 3.1). Transferability is further limited by ortholog-specific differences in seed region architecture, kinetic parameters, and sequence preferences across the Cas13 family [24,55].

No amount of architectural sophistication can rescue a model from hidden experimental variables. Current architectures encode guide sequence features but ignore parameters such as reporter molecule identity, even though ortholog-specific dinucleotide cleavage preferences mean that reporter choice alters signal output. Kinetic parameter estimates carry intrinsic uncertainty within a factor of 2 even for carefully executed experiments [45], and systematic errors in foundational datasets propagate to every downstream model regardless of network depth. Most published kinetic datasets fail basic validation criteria [38]. Authors rarely share raw time-series data, blocking independent verification, and most do not respond to data-sharing requests despite journal commitments [38]. The result is that models trained on one laboratory’s measurements absorb institution-specific reagent batches, buffer compositions, and measurement artifacts that are inseparable from true biological signal.

Accelerating methodological progress requires community-developed benchmarking infrastructure with specific structural elements. Without unified reference datasets and common metrics, numerical performance comparison across existing methods is still impossible [58]. Curated benchmark datasets must span multiple diagnostic Cas systems, encompassing the most commonly used orthologs *Lwa*Cas13a, *Lbu*Cas13a, *Lb*Cas12a, and *As*Cas12a. Pathogen coverage should extend across viral, bacterial, and eukaryotic targets. Sample matrix diversity must include blood, saliva, nasopharyngeal swabs, and urine. Sequence-similarity-based train/validation/test splits prevent data leakage. Core metrics including Spearman correlation (ρ), AUC, and limit-of-detection concordance are well established [58], yet cross-dataset performance degradation requires standardized reporting. Metadata recording reagent batches, buffer compositions, and instrument calibrations supports reproducibility and allows diagnosis of dataset-specific artifacts. Raw time-series fluorescence data with calibration standards must ideally be deposited in public repositories. Self-consistency checks based on mass conservation and reaction kinetics (α, β, γ parameters) identify grossly inconsistent data from published figures alone, permitting rapid quality assessment without requiring raw data access [38]. Without such infrastructure, the field cannot distinguish genuine algorithmic advances from artifacts of data scale or experimental design.

These data constraints expose one consequence of training on narrow laboratory conditions, namely that models absorb the biochemistry of a single experimental protocol and mistake it for general biology. A second consequence emerges when classifiers leave the laboratory entirely. Deployment introduces variability in hardware, lighting, temperature, and patient populations that no controlled training set anticipates, and validation rarely extends beyond the institution where a model was built.

### 4.2. Deployment Constraints in Signal Classification

Signal classification models achieve high accuracy under controlled conditions, yet three unresolved problems, confidence calibration, single-site validation bias, and environmental variability, separate laboratory performance from clinical deployment. The accuracies summarized in Table 3 are not in dispute. What those figures conceal is the absence of any framework for translating a model’s numerical output into a clinical decision. Most CRISPR-Dx classifiers employ inherently interpretable architectures (linear SVMs, logistic regression, decision tree ensembles), so interpretability is not the barrier. The barrier is that these classifiers output binary calls or regression scores without quantifying predictive uncertainty, and near-threshold samples demand exactly the kind of graded confidence that current systems cannot provide. A sample classified as positive at 51% probability warrants different clinical action from one classified at 99%, yet every published system collapses this range into a single binary output. Deep ensembles whose constituent networks output Beta-distributed predictions, parameterized by learned $\alpha_{0}$ and $\alpha_{1}$ coefficients that capture aleatoric uncertainty, have achieved precision exceeding 90% for Cas9 guide selection while maintaining greater than 93% gene coverage by flagging unreliable calls through interquartile-range thresholds [80]. These results show that uncertainty-aware architectures are operationally viable within CRISPR workflows, where graded confidence scores could replace the binary calls that currently dominate signal classification. Adapting such calibration to diagnostic signal classifiers, where the cost of a false negative can be a missed infection, remains an open and urgent engineering problem.

The validation evidence underlying these performance claims exposes a consistent and concerning pattern. Many signal classification models are trained or tuned on synthetic targets, purified samples, or small single-laboratory datasets under standardized conditions [62,66,72], and the studies that incorporate patient-derived specimens usually draw from single-site cohorts with narrow demographic and procedural diversity [68,73]. Performance on samples collected under different extraction protocols, from different patient populations, or at different viral loads is simply unknown. Among the nine classification-focused entries in Table 3, only COMPASS reports external validation on an independent cohort [74], and none report cross-laboratory reproducibility metrics. The pattern mirrors the data concentration problem in guide design, where reliance on a single dataset created systemic fragility across the predictive modeling field. For signal classifiers, single-institution validation creates an analogous vulnerability because the model absorbs site-specific confounders, including reagent batches, operator protocols, camera hardware, and patient demographics, that it cannot distinguish from genuine diagnostic signal. Until multi-site studies appear, reported accuracies function as upper bounds on real-world performance rather than estimates of clinical utility.

Environmental and hardware variability amplify the validation deficit, and pre-analytical variables take on particular force when classifiers leave the laboratory. Smartphone-based readout systems depend on ambient lighting, camera sensor response curves, and on-device computational capacity, parameters that vary unpredictably across deployment contexts [62,66]. LFA classifiers trained exclusively under indoor conditions with a restricted set of smartphone models have no demonstrated tolerance for the heterogeneity of field deployment. Temperature introduces a separate and more insidious problem. Isothermal amplification in resource-limited settings often relies on imprecise heating from chemical hand warmers [62,63], and ortholog selection alone produces a 23-fold sensitivity difference at room temperature for Cas12a [31]. Temperature-dependent activation kinetics ($k_{act}$) therefore alter assay signal intensity and temporal profile before the classifier ever processes an image, creating a confounding variable that no published model accounts for. No published study has systematically evaluated signal classifier performance across lighting conditions, temperature ranges, or device models, and the resulting deployment risk is still unquantified.

Closing these gaps requires interventions at the data, algorithmic, and hardware levels. Multi-site clinical datasets assembled from diverse patient populations, extraction protocols, and sample matrices (blood, saliva, nasopharyngeal swabs, urine) would expose models to the heterogeneity that single-site training conceals [5]. Cross-device validation protocols should mandate performance reporting across smartphone models, ambient lighting conditions, and temperature ranges, replacing the current practice of single-condition evaluation with benchmarks that reflect deployment reality. At the hardware level, clip-on optical adapters with controlled LED illumination and built-in calibration standards can suppress environmental variability before it reaches the classifier.

These deployment problems and the data constraints in guide design share a root cause, as data scarcity limits predictive models because narrow training sets cannot capture the biochemical diversity of clinical targets, and deployment variability limits signal classifiers because controlled-condition training cannot anticipate field heterogeneity. Training either class of model from scratch on small, laboratory-derived datasets will not yield representations that generalize to the clinic.

### 4.3. Potential of Transfer Learning and Foundation Models for CRISPR-Dx

No foundation model has been applied to CRISPR-Dx guide activity prediction; the connections drawn below between RNA language models and diagnostic design are extrapolations that await experimental testing. TIGER, EasyDesign, and ADAPT learn sequence motifs that correlate with Cas activity, but these CNN-based architectures degrade outside their training distributions. Existing diagnostic training sets sample too few pathogens, Cas orthologs, and reaction conditions to support clinical generalization. Foundation models (FM) trained on large biological sequence corpora offer an alternative by providing pretrained representations that could compensate for this scarcity (Figure 7). The structural and functional capabilities cited below are established within the domains where each model was benchmarked, whereas every claim that these representations improve CRISPR-Dx guide or trans-cleavage prediction remains untested.

Most DL research on CRISPR guide design has addressed genome editing, with Cas12a models predicting cis-cleavage efficiency [50] and Cas13d models optimizing guides for RNA knockdown [33,54]. Repurposing these editing-derived representations for diagnostics is complicated because editing models learn the determinants of cis-cleavage, a single irreversible double-strand break, whereas the diagnostic signal arises from multi-turnover collateral trans-cleavage, whose efficiency depends on catalytic activation steps beyond target binding. Diagnostic assays therefore optimize collateral catalytic turnover ($k_{cat}/K_{M}$) and must suppress false positives in complex clinical matrices, objectives absent from the single-cleavage events that editing models learn [40]; Section 4.4 develops this kinetic distinction. In a related sensing domain, DL architectures trained on toehold riboregulators generalized across RNA-based sensor platforms [82], providing indirect, non-diagnostic evidence that learned representations of RNA-mediated recognition can transfer between detection modalities, though no comparable transfer has been shown for Cas trans-cleavage.

Target accessibility represents the most plausible entry point for FM in CRISPR-Dx. Cas13 enzymes cleave single-stranded RNA preferentially, and secondary structures that occlude the protospacer region suppress activity [20,26]. The crRNA spacer contains functionally distinct subregions, a seed region governing target binding and a nuclease-switch region gating HEPN activation, yet sequence-based CNNs cannot determine whether a candidate target site is structurally accessible. Three RNA FM address this limitation at complementary levels of structural resolution. RiNALMo (650 M parameters, trained on 36 million non-coding RNA sequences) acquires general-purpose sequence representations that capture latent structural motifs and transfer across non-coding RNA prediction tasks including translation efficiency and expression level, capabilities benchmarked within those tasks rather than for CRISPR-Dx guide prediction [76]. For the secondary structure prediction most directly relevant to target accessibility, however, ERNIE-RNA outperforms RiNALMo despite being 7.5-fold smaller (86 M parameters) because it injects thermodynamic base-pairing priors (AU, GC, GU weights) directly into the transformer attention bias, achieving cross-family F1 scores approximately twice those of RiNALMo on held-out RNA families [77]. Neither model captures tertiary interactions that can bury a protospacer within three-dimensional folds. RhoFold+ fills this gap by combining a language model pretrained on approximately 23.7 million RNA sequences with multiple sequence alignment (MSA)-based co-evolutionary signals in an AlphaFold2-inspired architecture for end-to-end 3D structure prediction [78]. These accessibility and folding capabilities are established on RNA structure benchmarks, whereas the proposition that the resulting embeddings improve trans-cleavage prediction is a hypothesis, because no study has yet coupled any of these three FM to a CRISPR-Dx guide-activity readout. They span a representational hierarchy from general sequence embeddings through secondary structure accessibility to tertiary occlusion, offering structural resolution that current CNN-based guide design pipelines do not encode.

Experimental evidence that the pretrain-then-finetune paradigm works for RNA functional tasks now exists, though not yet in a diagnostic context. A modified nanoGPT architecture, trained on 80,789 bacterial and 4416 archaeal species from the GARNET database with overlapping triplet tokenization optimized for RNA, predicted thermostability-enhancing mutations in ribosomal RNA [79]. The predicted H92-CC mutations produced a 3-fold increase in ribosome activity at 65 °C upon experimental testing, confirming that the model had learned biophysically grounded representations. This thermostability result is experimentally confirmed evidence that RNA language models acquire transferable functional knowledge, yet it comes from a conservation-driven structural task rather than a diagnostic screen, so its extension to trans-cleavage guide activity remains an extrapolation.

Structural accessibility, however, accounts for only one dimension of guide efficacy. Seed-region complementarity, mismatch tolerance, and ortholog-specific activation kinetics all contribute, and these factors interact in ways that structural embeddings alone do not encode. FM-derived accessibility and folding features could serve as supplementary inputs to existing CNN architectures, reducing the feature engineering burden, but converting those features into accurate trans-cleavage predictions still requires diagnostic-specific fine-tuning data that remains scarce across effector families and pathogen targets. In the near term, augmenting current architectures with FM-derived features is more tractable than replacing them. Whether FM pretrained on evolutionary sequence diversity produce representations that transfer across Cas effector families will depend on closing the loop between computational prediction and experimental validation.

### 4.4. Toward Agentic AI and Reinforcement-Learning-Driven Design

Translating FM representations into clinically useful assays requires closed-loop systems that refine predictions through successive experimental cycles, learning from the failures that expose the limits of open-loop design.

BADGERS traverses guide sequence space through iterative mutation and CNN-based fitness scoring via the pretrained ADAPT model, yet neither system incorporates experimental outcomes to update its predictive weights [37,53]. When designed guides underperform, the cases that fail most dramatically, those encountering pathogens, matrices, or conditions absent from training data, are precisely where closed-loop correction would yield the greatest improvement. Agentic AI systems validated in adjacent domains establish architectural precedents for such correction. CRISPR-GPT coordinates large language model (LLM)-based planning with bioinformatics tool providers under human oversight across the full gene-editing workflow [83], while Robin closes the hypothesis–experiment cycle through integrated literature review, candidate ranking, and autonomous data analysis [84]. Autonomous agents have further demonstrated navigation of million-scale molecular search spaces [85]. The compositional tool integration of CRISPR-GPT and the hypothesis–feedback cycling of Robin together define the minimum architectural requirements for an agentic CRISPR-Dx system.

Adapting these architectures to diagnostics demands explicit encoding of the biological constraints governing detection. Sequence recognition motifs (PAM, PFS) and divergent seed architectures across Cas13 subtypes constrain the action space, requiring ortholog-specific positional weighting and hard filtering of non-compliant target sites [21,24,55]. Temperature-dependent $k_{act}$ variation and the exponential dependence of trans-cleavage rate on unwinding ΔG define physics-informed reward components that penalize kinetically unfavorable target sites under POC conditions [25,31,36]. Ortholog-specific dinucleotide cleavage preferences further transform guide design into multi-objective co-optimization of guide and reporter for the selected effector [35]. The same constraint logic extends to the readout half of the pipeline. LSTM-based kinetic analysis correlates temporal reaction features with target concentration more reliably than endpoint measurements [72], and a reinforcement learning (RL) agent controlling assay duration could learn when sufficient kinetic information has accumulated to issue a confident classification. The biomarker panels optimized by COMPASS and FEVOR exemplify combinatorial selection problems amenable to sequential decision-making, where each additional biomarker is chosen to maximize marginal diagnostic information gain [74,75]. End-to-end pipeline optimization, wherein a single agent jointly selects guide sequences, amplification conditions, reporter substrates, readout timing, and classification thresholds, represents the ultimate design target.

Model-based RL frameworks demonstrate that surrogate-assisted policy optimization efficiently controls biological sequence search under limited data [86], while evolutionary RL strategies discover high-fitness nucleic acid sequences without extensive labeled datasets [87]. Active learning offers the most compelling complement to RL for CRISPR-Dx, because rather than passively accumulating training data, an active policy strategically selects experiments that maximize information gain, prioritizing guide-target pairs in underrepresented regions of sequence space. Uncertainty-aware exploration could build on the deep ensemble architectures that achieve precision exceeding 96% for Cas9 guide selection with calibrated confidence estimates [80], routing guides with high ensemble disagreement to wet-lab testing and converting model failure into targeted data acquisition. Hybrid RL-mechanistic strategies incorporating experimental uncertainty into reward functions, building on work quantifying uncertainty in CRISPR kinetic parameters within a factor of two [45] and modeling reporter degradation as a first-order background process [46], offer reward signals grounded in measurement reality rather than idealized activity scores.

Lab-in-the-loop (LitL) approaches in adjacent domains yield compounding gains across iterative cycles. In therapeutic antibody optimization, four rounds of generative design coupled with multi-objective Bayesian optimization raised the hit rate for 3-fold-improved binders from 2% to 21% while simultaneously increasing model prediction accuracy (Spearman ρ from 0.35 to 0.54) [81]. This dual gain, better designs and better predictors, is the central property that separates LitL from one-shot training. Bayesian optimization has similarly guided brain-targeting delivery system design [88]. The AXIS system instantiates LitL principles in an imaging context, fine-tuning the DINOv2 vision FM through Low-Rank Adaptation (LoRA) and iterative expert curation to achieve 96.43% crystal recall [89]. The closest analogue in a CRISPR context reinforces this case: standard single-cell foundation models trained on observational transcriptomics generalize poorly to perturbation prediction, whereas closed-loop fine-tuning with perturbation data improves prediction of gene-expression changes [90]. The generalization failures documented in Section 3.1 provide the strongest motivation for LitL in CRISPR-Dx, because iterative experimental exposure to out-of-distribution conditions offers a mechanism for progressive distribution expansion that retraining on static data cannot achieve reliably.

These borrowed paradigms optimize objective functions that differ in kind from collateral trans-cleavage, and the distinction determines whether their metrics transfer. Antibody affinity maturation scores each variant by a single binding equilibrium, its dissociation constant or target occupancy, with no catalytic component [81], whereas collateral trans-cleavage is multi-turnover catalysis in which one target-activated complex cleaves thousands of reporters, making catalytic turnover ($k_{cat}\approx 27\;s^{-1}$, $k_{cat}/K_{M}$ up to $9.2\times 10^{7}\;M^{-1}\;s^{-1}$) rather than binding occupancy the diagnostic objective [29]. Genome editing optimizes cis-cleavage, the single on-target double-strand break, whereas the diagnostic signal depends on collateral trans-cleavage that on-target binding alone does not guarantee, and the link between binding and catalysis is itself ortholog-specific. In Cas13a the two are separable, since switch-region mismatches can tighten target binding 10- to 100-fold while abolishing HEPN nuclease activation entirely [24]; Cas12a lacks this HEPN architecture, and its trans-activation is gated instead by DNA-unwinding energetics, where a mismatch-sensing unwinding checkpoint licenses RuvC [25] and the logarithm of the trans-cleavage rate scales with the substrate strand-separation energy (−ΔG, Pearson r=0.94) [36]. Trans-cleavage is further shaped by temperature-dependent activation kinetics that shift ortholog sensitivity at point-of-care temperatures [31], by ortholog-specific dinucleotide reporter preferences [35], and by the need to suppress false-positive collateral activity in complex matrices, none of which appear in binding or editing objectives. The methodological machinery of lab-in-the-loop, agentic, and active-learning systems therefore transfers to CRISPR-Dx, but the objective function and the underlying kinetic model must be re-derived for multi-turnover collateral cleavage. Borrowed hit-rate and affinity metrics do not map onto diagnostic figures of merit such as limit of detection, catalytic efficiency, and the on-target to off-target collateral ratio, which is precisely why these proposals remain extrapolations rather than demonstrated results.

Realizing agentic and RL-driven CRISPR-Dx depends on infrastructure that does not yet exist. The benchmarking standards proposed in Section 4.1 must be in place before RL reward functions can be calibrated against reproducible experimental outcomes, and multi-site validation protocols are equally prerequisite because an RL agent trained on single-laboratory feedback will absorb institution-specific confounders just as static models do. Automated experimental platforms integrating liquid handling, real-time fluorescence monitoring, and ML-based signal classification must close the physical loop between computational proposals and wet-lab outcomes. With these prerequisites satisfied, the convergence of FM representations, agentic planning, and RL optimization points toward systems that propose, test, and iteratively refine complete diagnostic assays, compressing development timelines while maintaining the rigor that clinical deployment demands.

## 5. Conclusions

Machine learning has reshaped CRISPR-based diagnostics across the full pipeline, from guide RNA design and generation to automated point-of-care signal interpretation. The architectures perform well within their training distributions, but the data foundations beneath them are thin across both major effector families. Cas13a guide prediction depends on a single screening library; Cas12a fares worse, with one published diagnostic model and no high-throughput platform to expand its training data. Signal classification mirrors this concentration at the validation stage, as nearly all published models have been tested at single institutions on synthetic or narrowly sampled specimens. Models therefore absorb laboratory-specific confounders indistinguishable from biological signal, rendering reported accuracies upper bounds on clinical utility rather than estimates of it.

Beyond data scarcity, a representational gap separates current architectures from the biology they must predict. PAM and PFS recognition, divergent seed architectures, temperature-dependent activation kinetics, and ortholog-specific reporter preferences jointly define a design space whose dimensionality exceeds what sequence-trained networks capture. CNN architectures learn nucleotide correlations yet do not encode the physics and chemistry that determine whether a given guide produces reliable signal under field conditions.

Translating that vision into practice demands community infrastructure that does not yet exist. Shared benchmarks must span multiple Cas orthologs and clinical matrices, multi-site validation protocols must reflect deployment heterogeneity, and standardized kinetic data must enable reproducible comparison across laboratories. This infrastructure is a prerequisite for algorithmic progress rather than ancillary to it, because without shared standards the field cannot separate genuine methodological advances from artifacts of data scale or institutional protocol. 

## Figures and Tables

**Table 1:** Biochemical and kinetic parameters of Cas12a and Cas13 systems relevant to diagnostic assay design.

Parameter	Cas12a	Cas13
**Recognition**		
Target substrate	dsDNA	ssRNA
PAM/PFS	$5^{\prime }$-TTTN (canonical) ^**a**^	$3^{\prime }$ non-G PFS
Optimal spacer	20–24 nt	20–28 nt ^**b**^
**Specificity**		
Seed region	nt 1–6 (PAM-proximal)	nt 9–14 (central) ^**cd**^
HEPN switch	N/A	nt 5–8 (*Lbu*Cas13a) ^**c**^
Mismatch tolerance	Low in seed	Position-dependent
**Cleavage**		
Cis-cleavage	Required for activation	Not required ^**e**^
Trans substrate	ssDNA	ssRNA
Reporter	ssDNA (base-indifferent) ^**f**^	Ortholog-specific ^**f**^
Collateral activity	Lower ($k_{cat}$ 1–4 s^−1^)	Higher ($k_{cat}$ 17–740 s^−1^) ^**g**^
**Kinetics**		
$k_{cat}$ (trans)	1–4 s^−1^	∼27 s^−1^ (*Lbu*Cas13a) ^**g**^
$K_{M}$ (reporter)	20–50 nM	∼0.3 μM (*Lbu*Cas13a) ^**g**^
$k_{\mathrm{cat}}/K_{M}$	∼10^7^–10^8^ M^−1^s^−1^	∼10^7^–10^8^ M^−1^s^−1^ ^**g**^
Temp. optimum	37 °C	37 °C
**Sensitivity**		
LoD (amplified) ^**h**^	∼2 aM	∼2 aM
LoD (unamplified) ^**h**^	∼100 pM [32]	∼50 fM [35]

^**a**^ TTTV (V = A, C, or G) sites preferred; TTTT shows reduced cleavage efficiency [21]. *As*Cas12a shows expanded PAM recognition (TYCV, TATV; Y = C or T) with reduced activity; Cas13a orthologs prefer non-G at $3^{\prime }$ PFS. **^b^** Optimal spacer length for Cas13a orthologs is 28 nt [23,37]; Cas13d uses 23–30 nt [34]. **^c^** Central seed (nt 9–14) and HEPN-nuclease switch (nt 5–8) characterized in *Lbu*Cas13a; seed mismatches cause up to 47-fold decrease in binding affinity, while switch mismatches permit 10- to 100-fold tighter binding but abolish nuclease activity [24]. **^d^** Cas13d (*Rfx*Cas13d) seed architecture diverges from Cas13a [34]. **^e^** Cas13 trans-activation is coupled to target binding, not cleavage; target (cis) cleavage proceeds ∼5000-fold slower than trans-cleavage [29]. **^f^** *Lb*Cas12a shows minimal base preference (<2-fold) for ssDNA [29]; other orthologs not characterized. Cas13 reporter preferences: *Lwa*Cas13a (U-rich) [35], *Lbu*Cas13a (poly-U) [29], *Psm*Cas13b (GA), *Cca*Cas13b (UC) [11]. **^g^** Kinetic parameters from [29]; *Lbu*Cas13a efficiency ($9.2\times 10^{7}$ M^−1^s^−1^) approaches diffusion limit; Cas12a values validated [38]. **^h^** LoD with RPA/LAMP preamplification [6,7].

**Table 2:** Representative ML/DL tools for crRNA design applicable to CRISPR-based diagnostics.

Tool	Cas	Architecture	*n*	Performance (ρ)
DeepCpf1 [50]	12a	CNN + chromatin	15k	0.60–0.83 ^**a**^
EasyDesign [52]	12a	CNN	11k	0.79–0.84
ADAPT [37]	13a	CNN + optimization	19k	0.69 ^**b**^
Fu et al. [51]	13a	Dual-CNN + features	19k	0.70 ^**c**^
BADGERS [53]	13a	GAN (generative)	—	— ^**d**^
DeepCas13 [54]	13d	CNN-LSTM	6k	— ^**e**^
TIGER [55]	13d	CNN + features	200k	— ^**f**^

AUC, area under the receiver operating characteristic curve; AUPR, area under the precision–recall curve; CNN, convolutional neural network; GAN, generative adversarial network; LSTM, long short-term memory; *n*, approximate training set size (k = thousands); ρ, Spearman correlation on held-out test data; SNP, single-nucleotide polymorphism. Performance metrics vary by study design and are not directly comparable across different Cas systems or datasets. ^**a**^ Full model with chromatin accessibility features; sequence-only variant (Seq-deepCpf1) achieves lower correlation. ^**b**^ Primary test set (ρ = 0.687); external validation achieved ρ = 0.816. ^**c**^ Trained on the *Lwa*Cas13a dataset (same as ADAPT); cross-ortholog validation on 105k *Lbu*Cas13a binding-affinity pairs achieved ρ = 0.703. Classification AUC = 0.882 (*Lwa*Cas13a). ^**d**^ Generative approach achieving 10–20% sensitivity improvement and 2–3-fold higher specificity for SNP discrimination. ^**e**^ AUC = 0.87 on the held-out *CD46/55/71* five-fold cross-validation set; AUPR = 0.57 on an independent circRNA test set in HT29 cells [54], illustrating performance shifts across datasets and target classes. ^**f**^ Pearson *r* = 0.76 for off-target activity prediction; optimized for on/off-target discrimination.

**Table 3:** ML methods for CRISPR-Dx signal classification, quantification, and automated readout.

Study	Readout Type	ML Method ^e^	Task	Performance
Bao et al. [62]	Fluorescence	Linear SVM	Binary classification	LoD 1 fM; acc. ∼100%
MagicEye [63]	Colorimetric	CNN	Binary classification	Acc. 100% ^**a**^
Khosla et al. [72]	Fluorescence kinetics	LSTM RNN	Binary classification	Sens. 87%; spec. 100%; acc. 92.1%
Gao et al. [73]	Iontronic biosensor	Random Forest	Binary classification	Acc. 96.7%; AUC 0.998
COMPASS [74]	Cas12a fluorescence	Fully connected NN	Binary classification	AUC 0.954; acc. 89.6% ^**d**^
Xue et al. [66]	LFA (smartphone)	U-Net; MnUV3	Segmentation + classification	Acc. 96.5%; sens. 95.4%; spec. 97.6% ^**b**^
Li et al. [64]	Multi-RPA chip	YOLOv5-S	Object detection	mAP@0.5 0.996; LoD 1 aM
mutaSCAN [65]	High-throughput microfluidics	Fast R-CNN	Multi-class classification	Acc. 100% (>10 min) ^**f**^
FEVOR [75]	Cas13a fluorescence	LDA	Multi-class classification	AUC 0.940; sens. 91.7%; spec. 92.8% ^**c**^
Samacoïts et al. [68]	Fluorescence	Logistic regression	Quantification	AUC 0.93; acc. 95% (Ct < 33)
Zhao et al. [69]	Droplet images	Random Forest regression	Quantification	LoD 10 copies/μL
Xu et al. [70]	Dual-mode fluorescence	LASSO regression	Quantification	$R^{2}$ 0.995; LoD 4.6 fM
Xue et al. [71]	Colorimetric	Stacked ensemble	Quantification	$R^{2}$ 0.999

Acc., accuracy; AUC, area under the receiver operating characteristic curve; CNN, convolutional neural network; Ct, cycle threshold; LDA, linear discriminant analysis; LFA, lateral flow assay; LASSO, least absolute shrinkage and selection operator; LoD, limit of detection; LSTM, long short-term memory; mAP, mean average precision; NN, neural network; R-CNN, region-based convolutional neural network; RNN, recurrent neural network; sens., sensitivity; spec., specificity; SVM, support vector machine; U-Net, U-shaped convolutional network; YOLO, You Only Look Once. Performance metrics vary by task type and are not directly comparable across studies. **^a^** Achieved under optimal reporter conditions (ROX concentration >1 μM); performance degrades at lower concentrations. **^b^** Values shown are means across two architectures; U-Net and the MobileNetV3-derived MnUV3 model were evaluated independently. **^c^** Multiple binary classifiers for different clinical comparisons (normal vs. colorectal cancer (CRC), CRC vs. post-operative, normal vs. adenoma). **^d^** Classification distinguishes non-small cell lung cancer (NSCLC) from healthy using 9-aptamer proteome profile; external validation cohort. **^e^** Methods span a complexity spectrum from classical linear models (LDA, logistic regression, LASSO) through ensemble methods (Random Forest, stacked ensembles) to deep architectures (CNN, LSTM, YOLO, U-Net); all employ supervised learning from labeled data. **^f^** Three-class variant detection: wild-type positive, Omicron positive, and negative.

## Data Availability

No new data were created or analyzed in this study. Data sharing is not applicable to this article.

## Abbreviations

The following abbreviations are used in this manuscript:

AUC	area under the receiver operating characteristic curve
AUPR	area under the precision–recall curve
Cas	CRISPR-associated protein
circRNA	circular RNA
CNN	convolutional neural network
CRISPR	clustered regularly interspaced short palindromic repeats
CRISPR-Dx	CRISPR-based diagnostics
crRNA	CRISPR RNA
DL	deep learning
dsDNA	double-stranded DNA
FM	foundation model
HEPN	higher eukaryotes and prokaryotes nucleotide-binding
LAMP	loop-mediated isothermal amplification
LASSO	least absolute shrinkage and selection operator
LDA	linear discriminant analysis
LFA	lateral flow assay
LitL	lab-in-the-loop
LLM	large language model
LoD	limit of detection
LoRA	low-rank adaptation
LSTM	long short-term memory
mAP	mean average precision
miRNA	microRNA
ML	machine learning
MSA	multiple sequence alignment
NN	neural network
PAM	protospacer adjacent motif
PCR	polymerase chain reaction
PFS	protospacer flanking sequence
POC	point-of-care
R-CNN	region-based convolutional neural network
RL	reinforcement learning
RNN	recurrent neural network
RPA	recombinase polymerase amplification
RT-qPCR	reverse transcription quantitative PCR
sgRNA	single-guide RNA
SNP	single-nucleotide polymorphism
SNV	single-nucleotide variant
ssDNA	single-stranded DNA
ssRNA	single-stranded RNA
SVM	support vector machine
TAM	tag-adjacent mismatch
tracrRNA	trans-activating CRISPR RNA
U-Net	U-shaped convolutional network
WGAN-AM	Wasserstein generative adversarial network with activation maximization
YOLO	You Only Look Once
